# Musculoskeletal Pain, Physical Activity, Muscle Mass, and Mortality in Older Adults: Results from the Korean Longitudinal Study on Health and Aging (KLoSHA)

**DOI:** 10.3390/medicina60030462

**Published:** 2024-03-11

**Authors:** Sun-Woo Hwang, Chang-Woo Kim, Yun-Jeong Jang, Chang-Han Lee, Min-Kyun Oh, Ki-Woong Kim, Hak-Chul Jang, Jae-Young Lim, Se-Woong Chun, Seung-Kyu Lim

**Affiliations:** 1 Department of Rehabilitation Medicine, Gyeongsang National University College of Medicine, Gyeongsang National University Hospital, Jinju 52727, Republic of Korea; 2 Department of Rehabilitation Medicine, Gyeongsang National University College of Medicine, Gyeongsang National University Changwon Hospital, Changwon 51472, Republic of Korea; 3 Department of Neurospsychiatry, Seoul National University College of Medicine, Seoul National University Bundang Hospital, Seongnam 13620, Republic of Korea; 4 Department of Internal Medicine, Seoul National University College of Medicine, Seoul National University Bundang Hospital, Seongnam 13620, Republic of Korea; 5 Department of Rehabilitation Medicine, Seoul National University College of Medicine, Seoul National University Bundang Hospital, Seongnam 13620, Republic of Korea; 6 Department of Rehabilitation Medicine, Soonchunhyang University College of Medicine, Soonchunhyang University Cheonan Hospital, Cheonan 31151, Republic of Korea

**Keywords:** low back pain, musculoskeletal pain, exercise, muscle, skeletal, mortality

## Abstract

*Background and objectives:* Musculoskeletal (MSK) pain significantly impacts physical activity and quality of life in older adults, potentially influencing mortality. This study explored the relationship between MSK pain, physical activity, muscle mass, and mortality among older adults. *Material and Methods:* We studied 1000 participants in the Korean Longitudinal Study on Health and Aging (KLoSHA), a prospective, population-based cohort study of people aged 65 years or older. Survival status was tracked over a 5-year period. Correlations between low back pain (LBP), knee pain, regular exercise, appendicular skeletal muscle mass (ASM), and other variables were analyzed. Logistic regression analyses were used to identify independent risk factors for mortality. *Results:* Of the total participants, 829 (82.9%) survived over a 5-year period. Survivors tended to be younger, had a higher BMI, and were more active in regular exercise. In contrast, non-survivors exhibited a higher prevalence of both LBP and knee pain, along with increased instances of multiple MSK pains. Lower ASM correlated moderately with LBP and knee pain, whereas higher ASM was associated with regular exercise. There was a moderate correlation between LBP and knee pain, both of which were associated with a lack of regular exercise. Age, sex, ASM, and regular exercise were significant predictors, even though MSK pain itself did not directly predict all-cause mortality. *Conclusions:* This study demonstrated the independent association between ASM, regular exercise, and mortality. Although MSK pain did not directly correlate with all-cause mortality, the non-survivor group had higher levels of both single and multiple MSK pains. Recognizing the interplay of MSK pain, physical activity, and muscle mass for older adults, the research underscores the need for holistic strategies to enhance health outcomes in older individuals with MSK pain.

## 1. Introduction

Musculoskeletal (MSK) pain exacts a considerable toll on individuals’ physical activity levels and overall quality of life [1]. With a global prevalence of approximately 30% [2], the latest analysis of Global Burden of Disease (GBD) 2019 data revealed that around 1.71 billion people worldwide are affected by MSK conditions, such as low back pain (LBP), neck pain, fractures, other injuries, osteoarthritis (OA), amputation, and rheumatoid arthritis [3]. Notably, LBP and knee pain emerge as primary concerns, with LBP contributing significantly to the overall MSK burden, encompassing 570 million cases globally and accounting for 7.4% of global years lived with disability (YLDs). OA is also a major contributor, affecting 528 million people and causing 19 million YLDs, with the knee being the most frequently affected joint [3,4]. The prevalence of MSK pain rises notably with age [5], and its burden is anticipated to grow substantially alongside increasing global life expectancy [6], particularly affecting older adults, as over 20% of this population worldwide experiences LBP [7]. Remarkably, around 73% of individuals living with OA are aged over 55, and 60% are female [8].

In the context of MSK pain and mortality, the association between rheumatoid arthritis and increased mortality risk is well established [9]. However, the prevalence of this condition is only about 2% [10], and the relationship might differ when examining the broader spectrum of MSK pain. Recent meta-analyses examining back pain and knee pain have shown that adults with knee pain exhibited a notable 35–37% increased risk of reduced time-to-mortality [11]. In the context of back pain, a sex-specific pattern emerged, with an association between back pain and mortality observed in women and among men experiencing severe back pain. Conversely, no such correlation was found in men or those with non-severe back pain, though the meta-analysis underscored significant heterogeneity among the included studies [12].

The connection between MSK pain and mortality may not be immediately apparent, as it does not directly impact vital organs or internal health. However, MSK impairments affect muscles, bones, joints, and adjacent connective tissues, leading to limitations in mobility and physical activity [13]. Individuals with under-treated or prolonged MSK pain may gradually reduce the usage of the affected site to alleviate discomfort, potentially resulting in decreased muscle mass [14], the body’s largest endocrine organ, which is related to mortality [15]. Conversely, muscles affect pain perception [16], and physical activity can also influence pain perception [17] through various factors such as mood. This interplay underscores the multidimensional relationship between MSK pain, physical activity, muscle mass, and their collective impact on mortality.

Generally, reduced physical activity and decreased muscle mass are encompassed within the concept of frailty [18], and this frailty is known to increase the risk of negative health outcomes [19]. A study reviewing the association between frailty and MSK pain showed supportive results for their correlation; however, only twelve cross-sectional studies have more or less directly examined the relationship between frailty and pain [20]. Additionally, there is a lack of research evaluating the relationship between frailty, MSK pain, and mortality altogether.

This study aimed to investigate the relationship between MSK pain, physical activity, muscle mass, and mortality among community-dwelling older Koreans aged over 65 years. The findings provide insights into the intricate interplay of these factors, offering implications for clinical practice and strategies for managing the aging population.

## 2. Materials and Methods

### 2.1. Study Subjects

This study leveraged data from KLoSHA, a population-based prospective cohort in Seongnam, South Korea, targeting community-dwelling individuals aged 65 or older, selected via multistage, stratified probability sampling [21]. The study included adults aged 65 and older residing in Seongnam City, South Korea, as of 1 August 2005. The KLoSHA comprises two distinct cohorts: randomly sampled older adults aged 65 or older (Sample-RE) and voluntarily enrolled oldest-old individuals aged 85 or older (Sample-OO). To establish the Sample-RE, a simple random sample (*N* = 1118) was drawn from a roster of 61,730 individuals aged 65 years or older who were residents on 1 August 2005. Invitations to participate were extended through letters and telephone calls, with the sample selected using a computer-generated list of random numbers. For the Sample-OO, all residents aged 85 or older in Seongnam (*N* = 3166) were invited via letters and telephone calls. Those who declined participation after receiving detailed study information were excluded. The baseline assessment was conducted from September 2005 to September 2006, followed by a second wave five years later at Seoul National University Bundang Hospital (SNUBH). The baseline cohort comprised 1000 participants, including 441 men and 559 women. This study was approved by the Institutional Review Boards of SNUBH (B-0508/023-003), and written informed consent was obtained from all participants.

### 2.2. Demographics and Anthropometric Measures

Demographic characteristics were collected through medical history assessments and questionnaires administered by nurses certified in epidemiological studies and geriatric patient assessments. Weight and height were measured following the anthropometric standardization reference manual [22], recorded in centimeters and kilograms, and rounded to the nearest 0.1 unit. Body mass index (BMI) was calculated by dividing the total body weight (kg) by the height squared (m^2^). Waist circumference was measured horizontally at the narrowest part between the lowest rib and iliac crest. Appendicular skeletal muscle mass (ASM) was evaluated using a tetrapolar multi-frequency bioelectrical impedance analysis (Inbody 3; Biospace, Seoul, Korea), following a standardized procedure as previously reported [23]. This method has been validated against other body composition assessment techniques, including dual-energy X-ray absorptiometry, underwater weighing, and magnetic resonance imaging [24]. Participants undergo laboratory tests, including triglyceride, high-density lipoprotein (HDL) cholesterol, and fasting plasma glucose. Triglyceride and HDL cholesterol were enzymatically measured using an autoanalyzer (Hitachi 747; Hitachi, Ltd., Tokyo, Japan), and plasma glucose levels were determined by the glucose oxidase method. Blood pressure was measured twice in a sitting position using a standard mercury sphygmomanometer on a single occasion. Metabolic syndrome was defined as the concurrent presence of at least three out of the following five criteria [25]: (1) waist circumference equal to or exceeding 90 cm in men or 85 cm in women [26], (2) triglyceride levels measuring 150 mg/dL or higher, (3) high-density lipoprotein cholesterol falling below 40 mg/dL in men or 50 mg/dL in women, (4) blood pressure equal to or greater than 130/85 mmHg, or the use of antihypertensive medication, and (5) fasting plasma glucose at or above 100 mg/dL, or the use of pharmaceutical treatment for diabetes mellitus. We ascertained the mortality status of participants (survivor or non-survivor) when inviting them for the second-wave evaluation of the cohort study.

### 2.3. Knee Pain, Low Back Pain, and Regular Exercise

The questionnaire used in this study included the Western Ontario and McMaster Universities Osteoarthritis Index (WOMAC) for the knee [27], the Oswestry Low Back Pain Disability Questionnaire (ODQ) [28], and the International Physical Activity Questionnaire [29]. In our study, participants were classified as experiencing knee pain if their responses on the WOMAC questionnaire showed a score of 3 or higher on a 5-point Likert scale, specifically for knee pain at rest or during walking. Likewise, participants who scored 3 or higher for pain intensity or back pain during walking on the 6-point Likert scale within the ODQ were coded as having LBP. Finally, regular exercisers were defined as individuals engaging in moderate- or vigorous-intensity physical activities for 30 min or more on at least three occasions per week.

### 2.4. Statistical Analyses

Baseline characteristics were presented as mean ± standard deviation for continuous variables and as number of subjects (percentage) for categorical variables. We assessed differences between survivor and non-survivor groups using independent *t*-tests for continuous data and chi-squared tests for categorical data. Normal distribution was examined with the Kolmogorov–Smirnov and Shapiro–Wilk tests. The relationships between pain, regular exercise, and other variables were assessed using Cramer’s V or Phi correlation coefficients for nominal data, with significance determined by the chi-squared test. Correlations also examined individual pain types or the number of pain occurrences, regular exercise, and mortality. Simple logistic regression analyses were performed to identify potential predictors of death during the follow-up period. Variables with a *p*-value < 0.2 were subsequently included in multiple logistic regression models, employing a backward stepwise method to determine predictors of non-survival during the follow-up. All statistical analyses were conducted using IBM SPSS software (ver. 26.0; IBM Corp., Armonk, NY, USA). *p*-values < 0.05 were considered to indicate statistical significance.

## 3. Results

Of the 1000 older adults included in this study, 829 subjects (82.9%) had survived after a 5-year follow-up. The mean age differed significantly between the survivor and non-survivor groups, being 74.8 ± 8.1 and 83.7 ± 7.8 years, respectively (*p* < 0.001). The survivor group exhibited a higher BMI (*p* < 0.001). Additionally, the prevalence of LBP (*p* < 0.001) and knee pain (*p* = 0.016) was greater among non-survivors, while the proportion of regular exercisers was higher among survivors (*p* < 0.001). Factors such as sex, metabolic syndrome presence, alcohol consumption, and smoking did not differ significantly between the groups (Table 1).

**Figure 1:**
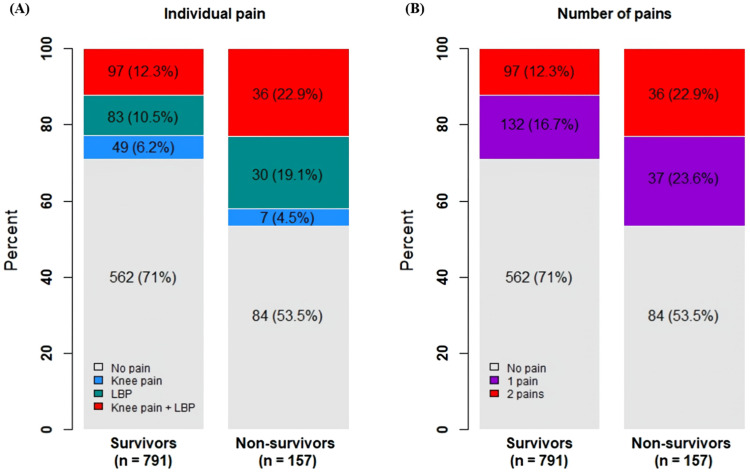
The proportion of MSK pain: (**A**) individual pain and (**B**) number of pains between survivor and non-survivor groups (n (%)). The non-survivor group showed significantly higher rates of MSK pain and multiple MSK pains.

The correlation between pain, regular exercise, and other variables was nearly significant, excluding BMI. Age exhibited a moderate association with LBP. Females showed moderate associations with LBP, knee pain, and regular exercise. Low ASM correlated moderately with LBP and knee pain, whereas high ASM correlated with regular exercise. LBP and knee pain had a moderate association with each other and with a lack of regular exercise (Table 2). The non-survivor group showed significantly higher rates of MSK pain and multiple MSK pains (Figure 1). Although correlations between pain occurrence and mortality were significant, the association was weak (Table 2).

Univariate analysis indicated a significant association between age, BMI, LBP, knee pain, MSK pain, regular exercise, and non-survivor status. Multiple logistic regression analyses were conducted using individual pain types or the number of pain occurrences as variables. Both regression models yielded consistent results: age (OR = 1.095, 95% confidence interval [CI] 1.062–1.126, *p* < 0.001) was associated with increased mortality risk, while female sex (OR = 0.138, 95% CI 0.063–0.302, *p* < 0.001), ASM (OR = 0.822, 95% CI 0.732–0.923, *p* = 0.001), and regular exercise (OR = 0.465, 95% CI 0.287–0.754, *p* < 0.002) were negatively associated with mortality. LBP, knee pain, and the number of MSK pain occurrences were not predictive of mortality in the multivariate models (Table 3).

## 4. Discussion

In this study, the non-survivor group exhibited higher rates of LBP and knee pain, whereas survivors were more often regular exercisers. Furthermore, the non-survivor group demonstrated increased levels of both individual and multiple MSK pains. Lower ASM correlated moderately with LBP and knee pain, whereas higher ASM was associated with regular exercise. LBP and knee pain were moderately correlated and associated with a lack of regular exercise. Age, sex, ASM, and regular exercise were significant predictors, even though MSK pain itself did not directly predict all-cause mortality over a period of up to 6 years.

The all-cause mortality rate in the KLoSHA cohort was 17.1%. Since we did not include variables such as comorbid conditions, the specific association between mortality and various factors remains uncertain. However, while MSK pain did not emerge as an independent predictor, aging seemed to exert a more significant influence on mortality outcomes, likely due to age-related comorbidities. Nevertheless, addressing MSK pain remains crucial given its elevated prevalence among older adults, ranging from 40% to 60% [30]. This type of pain poses significant challenges in managing older adults, impacting health outcomes related to falls, frailty, depression, and both cognitive and physical functional decline [31]. Additionally, multisite MSK pains affect a significant portion, ranging from 25% to 43%, of community-dwelling adults aged 65 years and older [32,33], contributing to increased disability in this age group [34]. This multisite pain is also associated with a more sedentary level of physical activity [35]. In our study, a higher proportion of older adults who did not survive reported experiencing both MSK pain and multisite MSK pains (22.9% vs. 12.3%) compared to survivors, suggesting a potentially higher risk of adverse health outcomes for this group.

LBP and knee pain are prevalent forms of MSK pain among older adults. Previous reviews suggest that such MSK pain might elevate mortality risks in older adults [11,12,36]. However, the potential pathways explaining this association remain unclear. Research based on large population cohort datasets revealed that the interference of MSK pain with daily activities, rather than just its presence or severity, significantly influences mortality rates [37]. A systematic review indicated that older adults with MSK pain tend to be less physically active compared to those without such pain [13]. Elevated concerns about pain and the risk of falling may lead these individuals to restrict activities that could exacerbate their pain or precipitate falls [38]. Additionally, MSK pain correlates directly with increased mobility limitations [39]. Patients with chronic LBP frequently exhibit limited physical performance, especially in joint movement and overall body mobility, often adopting positions that emphasize abdominal strength [40]. Furthermore, increased LBP severity corresponds with diminished lower-extremity muscle performance and slower gait speeds [41]. Knee pain has been shown to significantly decrease maximum gait speed [42] and notably compromise balance, thereby affecting levels of physical activity [43]. Ultimately, physical inactivity due to MSK pain could amplify the risk of chronic ailments and mortality [37,44].

Regular physical activity has been shown to offer significant health benefits. The 2018 Physical Activity Guidelines for Americans [45] recommend that adults should participate in at least 150 min of moderate-intensity aerobic activity or 75 min of vigorous-intensity aerobic activity each week, or a combination of both. Adults adhering to these guidelines showed a greatly reduced risk of all-cause and cause-specific mortality [46]. In this study, engaging in moderate- or vigorous-intensity physical activities for at least 30 min on a minimum of three occasions per week demonstrated a beneficial effect in reducing mortality rates, underscoring the importance of regular exercise. Although we did not treat these conditions as covariates, mental disorders such as cognitive impairment [47] or depression [48], cardiopulmonary conditions such as chronic respiratory diseases [49] or chronic heart failure [50], and anatomical factors such as kyphotic posture [51] are known to be associated with increased mortality. Considering our study results, the difficulty in engaging in regular physical activity due to these medical conditions may act as a confounding factor in the relationship between these factors and mortality.

BMI is known to be associated with all-cause mortality. In a large cohort study involving 3.6 million adults, a J-shaped association between BMI and all-cause mortality, with the lowest mortality at 25 kg/m^2^ was observed [52]. In our study, the average BMI of the survivor group was 24.1 kg/m^2^, higher than the deceased group’s average BMI of 22.9 kg/m^2^, classifying the survivor group as overweight and the deceased group as normal weight according to the World Health Organization’s Asia–Pacific region criteria [53]. There is a meta-analysis study showing that the mortality rate is lower in older adults who are obese or overweight compared to those in the normal weight group [54]. The inverse correlation between BMI and mortality is commonly explained as the obesity paradox. Among the hypotheses explaining this paradox, one suggests that a higher BMI is associated with enlarged muscle mass and better nutritional status, leading to a lower mortality rate [55]. In our study, while BMI showed a significant correlation with mortality in the univariate analysis, the fact that ASM, not BMI, was a predictor of mortality may support that hypothesis.

In our study, ASM emerged as an independent predictor of mortality and was associated with MSK pain. Skeletal muscles play a pivotal role in facilitating a wide spectrum of movements, ranging from powerful to delicate actions, underscoring their significance for physical performance [56]. As individuals advance in age, the inevitability of skeletal muscle atrophy becomes more pronounced, largely attributed to declining levels of testosterone, insulin-like growth factor-1 (IGF-1), and growth hormone. Contributing factors such as physical inactivity and malnutrition further exacerbate this muscle mass decline [57,58,59]. A recent review indicates that muscle mass in individuals aged 75 years or older declines at a rate of 0.64–0.70% per year in women and 0.80–0.98% per year in men, which is greater compared to younger age groups [60]. This age-related decline in muscle mass compromises physical performance [61], elevates the risk of physical disability in later life [62], and increases the overall mortality risk [63]. Additionally, levels of inflammatory cytokines are elevated in older adults compared to younger individuals [64]. These cytokines not only initiate but also sustain pathological pain by directly activating nociceptive sensory neurons [65]. Furthermore, they may hinder the differentiation of satellite cells, resulting in a gradual and progressive decline in muscle mass and quality [56]. As a consequence, older adults with reduced skeletal muscle mass may be more vulnerable to MSK pain.

When MSK pain in older adults remains inadequately managed, they often limit physical activity to mitigate discomfort or further pain. This avoidance or inability to engage in physical activities due to pain can lead to weight gain and exacerbate chronic conditions, thereby heightening mortality risks. Moreover, reduced physical activity contributes to disuse atrophy of skeletal muscles. Consequently, decreased muscle mass diminishes physical performance, leading to increased inactivity and elevated vulnerability to pain. The interplay between MSK pain, physical activity, and muscle mass can induce frailty in older adults, potentially increasing the health burden. In a meta-analysis investigating the relationship between frailty and health outcomes among community-dwelling older adults, frailty was found to increase the risk of negative health outcomes, including the loss of activities of daily living, physical limitations, falls and fractures, hospitalization, and mortality [19]. The relationships observed between pain, ASM, and regular exercise in this study underscore the importance of holistic treatment approaches for MSK pain. Prolonged rest strategies to manage pain should be avoided to avert muscle atrophy and declining physical activity. Along with pain management, patients should be encouraged to engage in exercises tailored to their MSK pain level. Neglecting to offer these recommendations would continue to exacerbate the negative mortality implications associated with pain. Furthermore, incorporating rehabilitative measures like exercise and nutritional support to enhance ASM could potentially reduce mortality rates.

Our study presents several noteworthy limitations. Firstly, the sample size was relatively small, potentially limiting our ability to identify additional significant predictors of mortality. A critical consideration involves the variable of physical activity, which relied on self-reported data, introducing the potential for bias. Additionally, we did not differentiate the severity of pain or exercise intensity, missing an opportunity to establish a compelling dose–response relationship. The assessment of MSK pain was based solely on our evaluation of prevalent and impactful LBP and knee pain in older adults, considering them as representative indicators. However, the limited generalizability of the results arises from not investigating MSK pain in various other commonly affected areas in older adults, potentially restricting the comprehensiveness of our findings. Furthermore, our analysis was restricted to baseline values, offering limited insights into changes over the study period that could clarify the causal sequence between variables and mortality. We also did not include confounding factors, such as nutritional status, cognitive impairment, depression, chronic respiratory disease, chronic heart failure, and kyphotic posture, as variables, despite their potential relevance to pain, physical activity, and mortality. Lastly, our examination was confined to all-cause mortality, without categorizing specific causes of death.

## 5. Conclusions

This study highlighted the independent association between ASM, regular exercise, and mortality. While MSK pain alone did not directly correlate with all-cause mortality, the non-survivor group showed elevated levels of both singular and multiple MSK pains. Recognizing the intricate relationship between MSK pain, physical activity, and muscle mass and their significance for older adults, this research emphasizes the necessity of comprehensive strategies. Such strategies should prioritize effective pain management and concurrently aim to maintain or enhance muscle mass and physical activity levels, thereby improving health outcomes in older individuals with MSK pain.

## Figures and Tables

**Table 1:** Characteristics of study participants.

	Total (*n* = 1000)	Survivor (*n* = 829)	Non-Survivor (*n* = 171)	*p*-Value
Age (years) (*n* = 1000)	76.3 ± 8.7	74.8 ± 8.1	83.7 ± 7.8	<0.001 *
Sex (*n* = 1000)				0.061
Male	441 (44.1)	354 (42.7)	87 (50.9)	
Female	559 (55.9)	475 (57.3)	84 (49.1)	
BMI (m^2^/kg) (*n* = 871)	24.0 ± 3.3	24.1 ± 3.3	22.9 ± 3.3	<0.001 *
ASM (kg) (*n* = 877)	12.8 ± 3.3	12.9 ± 3.3	12.3 ± 3.5	0.098
Metabolic syndrome (*n* = 996)				0.753
No	631 (63.4)	521 (63.1)	110 (64.7)	
Yes	365 (36.6)	305 (36.9)	60 (36.3)	
Low back pain (*n* = 950)				<0.001 *
No	703 (74.0)	612 (77.3)	91 (57.6)	
Yes	247 (26.0)	180 (22.7)	67 (42.4)	
Knee pain (*n* = 949)				0.016 *
No	760 (80.1)	645 (81.5)	115 (72.8)	
Yes	189 (19.9)	146 (18.5)	43 (27.2)	
Alcohol (*n* = 992)				0.185
No	761 (76.7)	625 (75.8)	136 (81.0)	
Yes	231 (23.3)	199 (24.2)	32 (19.0)	
Smoking (*n* = 1000)				1.000
No	711 (71.1)	589 (71.0)	122 (71.3)	
Yes	289 (28.9)	240 (29.0)	49 (28.7)	
Regular exercise (*n* = 989)				<0.001 *
No	492 (49.7)	377 (45.9)	115 (68.5)	
Yes	497 (50.3)	444 (54.1)	53 (31.5)	

Values are presented as mean ± standard deviation or number (%). BMI, body mass index; ASM, appendicular skeletal muscle mass; * significant difference between groups, *p* < 0.05 by independent *t*-test and chi-squared test.

**Table 2:** Correlations between pain, regular exercise, and other variables, as well as between specific pain types or pain occurrences and mortality.

	Variables		
Variables	Low Back Pain	Knee Pain	Regular Exercise
Age	0.220 **	0.160 **	0.152 **
Sex	0.255 **	0.279 **	0.291 **
BMI	−0.008	0.086 *	0.035
ASM	−0.245 **	−0.236 **	0.311 **
Metabolic syndrome	0.091 **	0.148 **	−0.069 **
Low back pain	-	0.506 **	−0.245 **
Knee pain	0.506 **	-	−0.264 **
Alcohol	−0.127 **	−0.176 **	0.129 **
Smoking	−0.122 **	−0.163 **	0.119 **
Regular exercise	−0.245 **	−0.264 **	-
	**Variables**		
**Variables**	Individual Pain ^†^	**MSK Pain** ^‡^	**Regular Exercise**
Mortality	0.164 **	0.145 **	−0.169 **

Values represent correlation coefficients (Cramer’s V, or Phi). BMI, body mass index; ASM, appendicular skeletal muscle mass; MSK, musculoskeletal. * *p* < 0.05; ** *p* < 0.01; ^†^ no pain, low back pain, knee pain, low back pain + knee pan; ^‡^ no pain, 1 pain (low back pain or knee pain), 2 pains (low back pain and knee pain).

**Table 3:** Results of logistic regression analysis of factors associated with non-survivors at the follow-up.

Characteristics	Univariate Model		Multivariate Model 1 ^§^		Multivariate Model 2 ^⁋^	
	OR (95% CI)	*p*	OR (95% CI)	*p*	OR (95% CI)	*p*
Age	1.133 (1.108–1.159)	<0.001	1.095 (1.065–1.126)	<0.001	1.095 (1.065–1.126)	<0.001
Sex (Female) *	0.720 (0.517–1.001)	0.051	0.138 (0.063–0.302)	<0.001	0.138 (0.063–0.302)	<0.001
Metabolic syndrome	0.932 (0.660–1.315)	0.688				
BMI	0.897 (0.894–1.010)	0.001		0.299		0.299
ASM	0.950 (0.383–0.751)	0.098	0.822 (0.732–0.923)	0.001	0.822 (0.732–0.923)	0.001
Low back pain	2.503 (1.752–3.576)	<0.001		0.442		
Knee pain	1.652 (1.114–2.499)	0.012		0.956		
Alcohol	0.739 (0.487–1.121)	0.155		0.235		0.235
Smoking	0.986 (0.685–1.418)	0.938				
Regular exercise	0.391 (0.275–0.557)	<0.001	0.465 (0.287–0.754)	0.002	0.465 (0.287–0.754)	0.002
MSK pain (1) ^†^	1.875 (1.219–2.885)	0.004				0.979
MSK pain (2) ^‡^	2.483 (1.590–3.879)	<0.001				0.661

BMI, body mass index; ASM, appendicular skeletal muscle mass; MSK, musculoskeletal; OR, odds ratio; CI, confidence interval. * Reference, male; ^†^ low back pain or knee pain; ^‡^ low back pain and knee pain; ^§^ including lower back pain and knee pain, respectively; **^⁋^** Including MSK pain categorized by number.

## Data Availability

The data presented in this study are available on request from the corresponding author.

## References

[1] Woolf A.D., Pfleger B. (2003). Burden of major musculoskeletal conditions. Bull. World Health Organ..

[2] Cimmino M.A., Ferrone C., Cutolo M. (2011). Epidemiology of chronic musculoskeletal pain. Best Pract. Res. Clin. Rheumatol..

[3] Cieza A., Causey K., Kamenov K., Hanson S.W., Chatterji S., Vos T. (2021). Global estimates of the need for rehabilitation based on the Global Burden of Disease study 2019: A systematic analysis for the Global Burden of Disease Study 2019. Lancet.

[4] (2023). Global, regional, and national burden of low back pain, 1990-2020, its attributable risk factors, and projections to 2050: A systematic analysis of the Global Burden of Disease Study 2021. Lancet Rheumatol..

[5] Fejer R., Ruhe A. (2012). What is the prevalence of musculoskeletal problems in the elderly population in developed countries? A systematic critical literature review. Chiropr. Man. Ther..

[6] Blyth F.M., Briggs A.M., Schneider C.H., Hoy D.G., March L.M. (2019). The global burden of musculoskeletal pain—Where to from here?. Am. J. Public Health.

[7] Briggs A.M., Cross M.J., Hoy D.G., Sànchez-Riera L., Blyth F.M., Woolf A.D., March L. (2016). Musculoskeletal health conditions represent a global threat to healthy aging: A report for the 2015 World Health Organization world report on ageing and health. Gerontologist.

[8] GBD 2019 Diseases and Injuries Collaborators (2020). Global burden of 369 diseases and injuries in 204 countries and territories, 1990–2019: A systematic analysis for the Global Burden of Disease Study 2019. Lancet.

[9] Sokka T., Abelson B., Pincus T. (2008). Mortality in rheumatoid arthritis: 2008 update. Clin. Exp. Rheumatol..

[10] Rasch E.K., Hirsch R., Paulose-Ram R., Hochberg M.C. (2003). Prevalence of rheumatoid arthritis in persons 60 years of age and older in the United States: Effect of different methods of case classification. Arthritis Rheum. Off. J. Am. Coll. Rheumatol..

[11] Leyland K.M., Gates L.S., Sanchez-Santos M.T., Nevitt M.C., Felson D., Jones G., Jordan J.M., Judge A., Prieto-Alhambra D., Yoshimura N. (2021). Knee osteoarthritis and time-to all-cause mortality in six community-based cohorts: An international meta-analysis of individual participant-level data. Aging Clin. Exp. Res..

[12] Roseen E.J., Rajendran I., Stein P., Fredman L., Fink H.A., LaValley M.P., Saper R.B. (2021). Association of back pain with mortality: A systematic review and meta-analysis of cohort studies. J. Gen. Intern. Med..

[13] Stubbs B., Binnekade T.T., Soundy A., Schofield P., Huijnen I.P., Eggermont L.H. (2013). Are older adults with chronic musculoskeletal pain less active than older adults without pain? A systematic review and meta-analysis. Pain Med..

[14] Baumgartner R.N., Waters D.L., Gallagher D., Morley J.E., Garry P.J. (1999). Predictors of skeletal muscle mass in elderly men and women. Mech. Ageing Dev..

[15] Chuang S.-Y., Chang H.-Y., Lee M.-S., Chen R.C.-Y., Pan W.-H. (2014). Skeletal muscle mass and risk of death in an elderly population. Nutr. Metab. Cardiovasc. Dis..

[16] Pan F., Tian J., Scott D., Cicuttini F., Jones G. (2022). Muscle function, quality, and relative mass are associated with knee pain trajectory over 10.7 years. Pain.

[17] Law L.F., Sluka K.A. (2017). How does physical activity modulate pain?. Pain.

[18] Xue Q.L. (2011). The frailty syndrome: Definition and natural history. Clin. Geriatr. Med..

[19] Vermeiren S., Vella-Azzopardi R., Beckwée D., Habbig A.K., Scafoglieri A., Jansen B., Bautmans I. (2016). Frailty and the Prediction of Negative Health Outcomes: A Meta-Analysis. J. Am. Med. Dir. Assoc..

[20] Nessighaoui H., Lilamand M., Patel K.V., Vellas B., Laroche M.L., Dantoine T., Cesari M. (2015). Frailty and Pain: Two Related Conditions. J. Frailty Aging.

[21] Huh Y., Yang E.J., Lee S.A., Lim J.Y., Kim K.W., Paik N.J. (2011). Association between executive function and physical performance in older Korean adults: Findings from the Korean Longitudinal Study on Health and Aging (KLoSHA). Arch. Gerontol. Geriatr..

[22] Lohman T.G., Roche A.F., Martorell R. (1988). Anthropometric Standardization Reference Manual.

[23] Lukaski H.C., Johnson P.E., Bolonchuk W.W., Lykken G.I. (1985). Assessment of fat-free mass using bioelectrical impedance measurements of the human body. Am. J. Clin. Nutr..

[24] Mijnarends D.M., Meijers J.M., Halfens R.J., ter Borg S., Luiking Y.C., Verlaan S., Schoberer D., Cruz Jentoft A.J., van Loon L.J., Schols J.M. (2013). Validity and reliability of tools to measure muscle mass, strength, and physical performance in community-dwelling older people: A systematic review. J. Am. Med. Dir. Assoc..

[25] Expert Panel on Detection, Evaluation, and Treatment of High Blood Cholesterol in Adults (2001). Executive Summary of The Third Report of The National Cholesterol Education Program (NCEP) Expert Panel on Detection, Evaluation, and Treatment of High Blood Cholesterol in Adults (Adult Treatment Panel III). JAMA.

[26] Lee S.Y., Park H.S., Kim D.J., Han J.H., Kim S.M., Cho G.J., Kim D.Y., Kwon H.S., Kim S.R., Lee C.B. (2007). Appropriate waist circumference cutoff points for central obesity in Korean adults. Diabetes Res. Clin. Pract..

[27] Bellamy N., Buchanan W.W., Goldsmith C.H., Campbell J., Stitt L.W. (1988). Validation study of WOMAC: A health status instrument for measuring clinically important patient relevant outcomes to antirheumatic drug therapy in patients with osteoarthritis of the hip or knee. J. Rheumatol..

[28] Fairbank J.C., Couper J., Davies J.B., O’Brien J.P. (1980). The Oswestry low back pain disability questionnaire. Physiotherapy.

[29] Craig C.L., Marshall A.L., Sjöström M., Bauman A.E., Booth M.L., Ainsworth B.E., Pratt M., Ekelund U., Yngve A., Sallis J.F. (2003). International physical activity questionnaire: 12-country reliability and validity. Med. Sci. Sports Exerc..

[30] Dahlhamer J., Lucas J., Zelaya C., Nahin R., Mackey S., DeBar L., Kerns R., Von Korff M., Porter L., Helmick C. (2018). Prevalence of Chronic Pain and High-Impact Chronic Pain Among Adults—United States, 2016. MMWR Morb. Mortal. Wkly. Rep..

[31] Blyth F.M., Noguchi N. (2017). Chronic musculoskeletal pain and its impact on older people. Best Pract. Res. Clin. Rheumatol..

[32] Eggermont L.H., Bean J.F., Guralnik J.M., Leveille S.G. (2009). Comparing pain severity versus pain location in the MOBILIZE Boston study: Chronic pain and lower extremity function. J. Gerontol. A Biol. Sci. Med. Sci..

[33] Croft P., Jordan K., Jinks C. (2005). “Pain elsewhere” and the impact of knee pain in older people. Arthritis Rheum..

[34] Rundell S.D., Patel K.V., Krook M.A., Heagerty P.J., Suri P., Friedly J.L., Turner J.A., Deyo R.A., Bauer Z., Nerenz D.R. (2019). Multi-site Pain Is Associated with Long-term Patient-Reported Outcomes in Older Adults with Persistent Back Pain. Pain Med..

[35] de Vitta A., Machado Maciel N., Bento T.P.F., Genebra C., Simeão S. (2022). Multisite musculoskeletal pain in the general population: A cross-sectional survey. Sao Paulo Med. J..

[36] Chen L., Ferreira M.L., Nassar N., Preen D.B., Hopper J.L., Li S., Bui M., Beckenkamp P.R., Shi B., Arden N.K. (2021). Association of chronic musculoskeletal pain with mortality among UK adults: A population-based cohort study with mediation analysis. EClinicalMedicine.

[37] Patterson R., McNamara E., Tainio M., de Sá T.H., Smith A.D., Sharp S.J., Edwards P., Woodcock J., Brage S., Wijndaele K. (2018). Sedentary behaviour and risk of all-cause, cardiovascular and cancer mortality, and incident type 2 diabetes: A systematic review and dose response meta-analysis. Eur. J. Epidemiol..

[38] Martin R.R., Hadjistavropoulos T., McCreary D.R. (2005). Fear of pain and fear of falling among younger and older adults with musculoskeletal pain conditions. Pain Res. Manag..

[39] Karttunen N., Lihavainen K., Sipilä S., Rantanen T., Sulkava R., Hartikainen S. (2012). Musculoskeletal pain and use of analgesics in relation to mobility limitation among community-dwelling persons aged 75 years and older. Eur. J. Pain.

[40] Panhale V.P., Gurav R.S., Nahar S.K. (2016). Association of Physical Performance and Fear-Avoidance Beliefs in Adults with Chronic Low Back Pain. Ann. Med. Health Sci. Res..

[41] Leveille S.G., Guralnik J.M., Hochberg M., Hirsch R., Ferrucci L., Langlois J., Rantanen T., Ling S. (1999). Low back pain and disability in older women: Independent association with difficulty but not inability to perform daily activities. J. Gerontol. A Biol. Sci. Med. Sci..

[42] Kitayuguchi J., Kamada M., Hamano T., Nabika T., Shiwaku K., Kamioka H., Okada S., Mutoh Y. (2016). Association between knee pain and gait speed decline in rural Japanese community-dwelling older adults: 1-year prospective cohort study. Geriatr. Gerontol. Int..

[43] Yanardag M., Şimşek T.T., Yanardag F. (2021). Exploring the Relationship of Pain, Balance, Gait Function, and Quality of Life in Older Adults with Hip and Knee Pain. Pain Manag. Nurs..

[44] Biswas A., Oh P.I., Faulkner G.E., Bajaj R.R., Silver M.A., Mitchell M.S., Alter D.A. (2015). Sedentary time and its association with risk for disease incidence, mortality, and hospitalization in adults: A systematic review and meta-analysis. Ann. Intern. Med..

[45] Piercy K.L., Troiano R.P., Ballard R.M., Carlson S.A., Fulton J.E., Galuska D.A., George S.M., Olson R.D. (2018). The Physical Activity Guidelines for Americans. JAMA.

[46] Zhao M., Veeranki S.P., Magnussen C.G., Xi B. (2020). Recommended physical activity and all cause and cause specific mortality in US adults: Prospective cohort study. BMJ.

[47] Lin S., Chen M. (2022). Gender-specific impact of cognitive impairment on all-cause mortality in older persons: A meta-analysis. Exp. Gerontol..

[48] Walker E.R., McGee R.E., Druss B.G. (2015). Mortality in mental disorders and global disease burden implications: A systematic review and meta-analysis. JAMA Psychiatry.

[49] Soriano J.B., Kendrick P.J., Paulson K.R., Gupta V., Abrams E.M., Adedoyin R.A., Adhikari T.B., Advani S.M., Agrawal A., Ahmadian E. (2020). Prevalence and attributable health burden of chronic respiratory diseases, 1990–2017: A systematic analysis for the Global Burden of Disease Study 2017. Lancet Respir. Med..

[50] Jones N.R., Roalfe A.K., Adoki I., Hobbs F.D.R., Taylor C.J. (2019). Survival of patients with chronic heart failure in the community: A systematic review and meta-analysis. Eur. J. Heart Fail..

[51] Hijikata Y., Kamitani T., Sekiguchi M., Otani K., Konno S.I., Takegami M., Fukuhara S., Yamamoto Y. (2022). Association of kyphotic posture with loss of independence and mortality in a community-based prospective cohort study: The Locomotive Syndrome and Health Outcomes in Aizu Cohort Study (LOHAS). BMJ Open.

[52] Bhaskaran K., Dos-Santos-Silva I., Leon D.A., Douglas I.J., Smeeth L. (2018). Association of BMI with overall and cause-specific mortality: A population-based cohort study of 3·6 million adults in the UK. Lancet Diabetes Endocrinol..

[53] World Health Organization (2000). The Asia-Pacific Perspective: Redefining Obesity and Its Treatment.

[54] Veronese N., Cereda E., Solmi M., Fowler S.A., Manzato E., Maggi S., Manu P., Abe E., Hayashi K., Allard J.P. (2015). Inverse relationship between body mass index and mortality in older nursing home residents: A meta-analysis of 19,538 elderly subjects. Obes. Rev..

[55] Ades P.A., Savage P.D. (2010). The obesity paradox: Perception vs knowledge. Mayo Clin. Proc..

[56] Tieland M., Trouwborst I., Clark B.C. (2018). Skeletal muscle performance and ageing. J. Cachexia Sarcopenia Muscle.

[57] Nguyen C.P., Hirsch M.S., Moeny D., Kaul S., Mohamoud M., Joffe H.V. (2015). Testosterone and “Age-Related Hypogonadism”—FDA Concerns. N. Engl. J. Med..

[58] Hermann M., Berger P. (2001). Hormonal changes in aging men: A therapeutic indication?. Exp. Gerontol..

[59] Curcio F., Ferro G., Basile C., Liguori I., Parrella P., Pirozzi F., Della-Morte D., Gargiulo G., Testa G., Tocchetti C.G. (2016). Biomarkers in sarcopenia: A multifactorial approach. Exp. Gerontol..

[60] Mitchell W.K., Williams J., Atherton P., Larvin M., Lund J., Narici M. (2012). Sarcopenia, dynapenia, and the impact of advancing age on human skeletal muscle size and strength; a quantitative review. Front. Physiol..

[61] Wang D.X.M., Yao J., Zirek Y., Reijnierse E.M., Maier A.B. (2020). Muscle mass, strength, and physical performance predicting activities of daily living: A meta-analysis. J. Cachexia Sarcopenia Muscle.

[62] Berger M.J., Doherty T.J. (2010). Sarcopenia: Prevalence, mechanisms, and functional consequences. Interdiscip. Top. Gerontol..

[63] Zhou H.H., Liao Y., Peng Z., Liu F., Wang Q., Yang W. (2023). Association of muscle wasting with mortality risk among adults: A systematic review and meta-analysis of prospective studies. J. Cachexia Sarcopenia Muscle.

[64] Stowe R.P., Peek M.K., Cutchin M.P., Goodwin J.S. (2010). Plasma cytokine levels in a population-based study: Relation to age and ethnicity. J. Gerontol. A Biol. Sci. Med. Sci..

[65] Zhang J.M., An J. (2007). Cytokines, inflammation, and pain. Int. Anesthesiol. Clin..

